# A novel anti-EGFR monoclonal antibody (EMab-17) exerts antitumor activity against oral squamous cell carcinomas via antibody-dependent cellular cytotoxicity and complement-dependent cytotoxicity

**DOI:** 10.3892/ol.2020.11384

**Published:** 2020-02-10

**Authors:** Junko Takei, Mika Kato Kaneko, Tomokazu Ohishi, Manabu Kawada, Hiroyuki Harada, Yukinari Kato

**Affiliations:** 1 Department of Antibody Drug Development, Tohoku University Graduate School of Medicine, Aoba-ku, Sendai, Miyagi 980-8575, Japan; 2 Department of Oral and Maxillofacial Surgery, Graduate School of Medical and Dental Sciences, Tokyo Medical and Dental University, Bunkyo-ku, Tokyo 113-8510, Japan; 3 Institute of Microbial Chemistry (BIKAKEN), Numazu, Microbial Chemistry Research Foundation, Numazu-shi, Shizuoka 410-0301, Japan; 4 New Industry Creation Hatchery Center, Tohoku University, Aoba-ku, Sendai, Miyagi 980-8575, Japan

**Keywords:** epidermal growth factor receptor, monoclonal antibody, antibody-dependent cellular cytotoxicity, complement-dependent cytotoxicity, oral squamous cell carcinoma

## Abstract

The epidermal growth factor receptor (EGFR) is a member of the human epidermal growth factor receptor (HER) family of receptor tyrosine kinases; it is a transmembrane receptor involved in cell growth and differentiation. EGFR homodimers or heterodimers in combination with other HER members, such as HER2 and HER3, activate downstream signaling cascades in many types of cancer, including oral squamous cell carcinoma (OSCC). The present study produced novel anti-EGFR monoclonal antibodies (mAbs) possessing antibody-dependent cellular cytotoxicity (ADCC) and complement-dependent cytotoxicity (CDC), and investigated antitumor activity. Mice were immunized with an EGFR-overexpressed glioblastoma cell line, LN229 (LN229/EGFR), after which ELISA was performed using recombinant EGFR. mAbs were subsequently selected according to their efficacy for LN229/EGFR, as determined via flow cytometry. After determining the subclass of mAbs, the EMab-17 (IgG_2a_, kappa) clone exhibited ADCC and CDC activities against two OSCC cell lines, HSC-2 and SAS. Furthermore, EMab-17 exerted antitumor activities against mouse xenograft models using HSC-2 and SAS, indicating that EMab-17 may be used in an antibody-based therapy for EGFR-expressing OSCC.

## Introduction

Oral squamous cell carcinoma (OSCC) is usually treated by surgical removal; it can be complemented by chemotherapy, including cisplatin (CDDP), 5-fluorouracil (5-FU), and docetaxel (1,2), and/or radiotherapy, particularly at advanced stages. As an antibody drug, cetuximab, which is a mouse-human (IgG_1_) chimeric antibody against the epidermal growth factor receptor (EGFR), was approved for the treatment of head and neck cancer (HNC), including oral cancer (1). The effectiveness of cetuximab against locoregionally advanced head and neck squamous cell carcinoma (HNSCC) or recurrent and/or metastatic (R/M) HNSCC was reported in various clinical studies (1,3–5). Recently, nivolumab-a fully human IgG_4_ monoclonal antibody (mAb) against programmed cell death-1 (PD-1)-was approved for the treatment of R/M HNC previously treated with platinum-based chemotherapy (6). Furthermore, bevacizumab, which is a mouse-human IgG_1_ chimeric antibody against vascular endothelial growth factor first approved for colorectal cancer treatment, was the subject of clinical trials involving R/M HNSCC patients (7). Molecular targeting drugs that are clinically applicable for oral cancers are limited; therefore, novel drugs with greater efficacy and lower toxicity are required.

EGFR is a member of the human epidermal growth factor receptor (HER) family of receptor tyrosine kinases and involved in cell growth and differentiation (8–10). EGFR homodimers or heterodimers in conjunction with other HER members (such as HER2 and HER3) activate downstream signaling cascades. These pathways are frequently dysregulated via the overexpression of EGFR in many malignant tumors, including colorectal, lung, and breast cancers, brain tumors, head and neck cancers, pancreatic, kidney, and prostate cancers, and ovarian, bladder, and oral cancers (11).

In the previous research of this study, mice were immunized with purified recombinant EGFR to produce an EMab-134 monoclonal antibody (mAb; IgG_1_, kappa), which reacted with the endogenous EGFR of oral cancers in flow cytometry, Western blotting, and immunohistochemistry (12). In immunohistochemical analysis, EMab-134 stained 36 of 38 (94.7%) oral cancer specimens. The minimum epitope of EMab-134 was found to be the _377-_RGDSFTHTPP_−386_ sequence (13). Although EMab-134 is a very useful mAb-targeting EGFR, the subclass was determined to be mouse IgG_1_, which did not exhibit antibody-dependent cellular cytotoxicity (ADCC) and complement-dependent cytotoxicity (CDC) activities. This study develops novel anti-EGFR mAbs possessing the ADCC and CDC activities of mouse IgG_2a_ or the IgG_2b_ subclass and investigates the antitumor activity.

## Materials and methods

### 

#### Cell lines

HSC-2 and SAS were obtained from the Japanese Collection of Research Bioresources Cell Bank. Chinese hamster ovaries (CHO)-K1, P3X63Ag8U.1 (P3U1), and LN229 were obtained from the American Type Culture Collection. LN229/EGFR (a stable transfectant) was previously produced by transfecting pCAG/PA-EGFR-RAP-MAP (14) into LN229 cells using the Neon Transfection System (Thermo Fisher Scientific, Inc.), and EGFR upregulation was demonstrated by Western blot analysis using anti-EGFR mAb, clone EMab-51 (15). P3U1 was cultured in Roswell Park Memorial Institute (RPMI) 1640 medium (Nacalai Tesque, Inc.), while LN229, LN229/EGFR, HSC-2, and SAS were cultured in Dulbecco's Modified Eagle's medium (DMEM) (Nacalai Tesque, Inc.) supplemented with 10% heat-inactivated fetal bovine serum (FBS) (Thermo Fisher Scientific, Inc.), 100 units/ml of penicillin, 100 µg/ml of streptomycin, and 25 µg/ml of amphotericin B (Nacalai Tesque, Inc.) at 37°C in a humidified atmosphere containing 5% CO_2_ and 95% air.

#### Animals

All animal experiments were performed in accordance with relevant guidelines and regulations to minimize animal suffering and distress in the laboratory. Animal experiments described in the hybridoma production were approved by the Animal Care and Use Committee of Tohoku University (Permit number: 2016MdA-153). Mice were monitored for health every day. Animal studies for the antitumor activity were approved by the institutional committee for experiments of the Institute of Microbial Chemistry (Permit number: 2019-014). Mice were monitored for health and weight every 3 or 4 days. The duration of the experiment was three weeks. A body weight loss exceeding 25% of total body weight and a maximum tumor size exceeding 3,000 mm^3^ were defined as a humane endpoint.

#### Hybridoma production

One four-week-old female BALB/c mouse was purchased from CLEA Japan and housed under specific pathogen-free conditions. Anti-EGFR hybridomas were produced, as previously mentioned (15). The ectodomain of EGFR with N-terminal PA tag (16), C-terminal RAP tag (17), and MAP tag (14) (EGFRec) was purified from the supernatant of LN229/EGFRec using the anti-RAP tag previously described (17).

One BALB/c mouse was immunized by intraperitoneal injections of LN229/EGFR together with Imject Alum (Thermo Fisher Scientific, Inc.). After several additional immunizations, a booster injection was intraperitoneally administered 2 days before harvesting spleen cells. Spleen cells were then fused with P3U1 cells using GenomONE-CF (Ishihara Sangyo Kaisha, Ltd.). The resulting hybridomas were cultured in an RPMI medium supplemented with hypoxanthine, aminopterin, and thymidine selection medium supplement (Thermo Fisher Scientific, Inc.). Culture supernatants were screened using enzyme-linked immunosorbent assays (ELISA) with a recombinant EGFR-extracellular domain. mAbs were purified from the supernatants of hybridomas, cultured in Hybridoma-SFM medium (Thermo Fisher Scientific, Inc.) using Protein G Sepharose 4 Fast Flow (GE Healthcare UK, Ltd.).

#### Enzyme-linked immunosorbent assay

Recombinant proteins were immobilized on Nunc MaxiSorp 96-well immunoplates (Thermo Fisher Scientific, Inc.) at 1 µg/ml for 30 min. After blocking using a SuperBlock buffer (Thermo Fisher Scientific Inc.), the plates were incubated with primary antibodies, followed by 1:2,000 diluted peroxidase-conjugated anti-mouse IgG (Agilent Technologies). The enzymatic reaction was produced using a 1-Step Ultra TMB-ELISA (Thermo Fisher Scientific, Inc.). The optical density was measured at 655 nm using an iMark microplate reader (Bio-Rad Laboratories, Inc.).

#### Flow cytometry

Cells were harvested by brief exposure to 0.25% trypsin/1-mM ethylenediaminetetraacetic acid (EDTA) (Nacalai Tesque, Inc.). The cells were washed with 0.1% bovine serum albumin (BSA)/phosphate-buffered saline (PBS) and treated with 1 µg/ml of anti-EGFR mAbs for 30 min at 4°C, followed by Alexa Fluor 488-conjugated anti-mouse IgG (1:1,000; Cell Signaling Technology, Inc.). Fluorescence data was collected using EC800 Cell Analyzers (Sony Corp.).

#### Determination of the binding affinity using flow cytometry

SAS (2×10^5^ cells) was suspended in 100 µl of serially diluted mAbs (6 ng/ml-100 µg/ml); Alexa Fluor 488-conjugated anti-mouse IgG (1:1,000) (Cell Signaling Technology, Inc.) was then added, and fluorescence data was collected using a cell analyzer (EC800) (Sony Corp.). The dissociation constants (*K*_D_) were computed by fitting the binding isotherms using the built-in one-site binding models in GraphPad Prism 6 (GraphPad Software, Inc.).

#### ADCC

Six six-week-old female BALB/c nude mice were purchased from Charles River, and spleens were removed aseptically, and single-cell suspensions were obtained by dispersing the spleens using a syringe and pressing through stainless steel mesh. Erythrocytes were effectively lysed by 10-s exposure to ice-cold distilled water. Splenocytes were washed with DMEM and resuspended in DMEM with 10% FBS as effector cells. Target cells were labeled with 10-µg/ml Calcein AM (Thermo Fisher Scientific, Inc.) and resuspended in the medium. The target cells (2×10^4^ cells/well) were placed in 96-well plates and mixed with effector cells, anti-EGFR antibodies, or control IgG (mouse IgG_2a_) (Sigma-Aldrich Corp.). After a 4-h incubation period, the Calcein AM release of supernatant from each well was measured. The fluorescence intensity was determined at an excitation wavelength of 485 nm and an emission wavelength of 538 nm using a microplate reader (Power Scan HT) (BioTek Instruments). Cytolytic activity (as % of lysis) was calculated using the following formula: % lysis=(E-S)/(M-S) ×100 (where E is the fluorescence released in the experimental cultures of target and effector cells, S is the spontaneous fluorescence released in cultures containing only target cells, and M is the maximum fluorescence obtained by adding a lysis buffer containing 0.5% Triton X-100, 10 mM Tris-HCl (pH 7.4), and 10 mM of EDTA to the target cells in order to lyse all cells).

#### CDC

HSC-2 and SAS cells were placed in 96-well plates of 2×10^4^ cells/well in DMEM supplemented with 10% FBS. Cells were incubated with either anti-EGFR antibodies or the control IgG (mouse IgG_2a_) (Sigma-Aldrich Corp.) and 10% of rabbit complement (Low-Tox-M Rabbit Complement) (Cedarlane Laboratories) for 5 h at 37°C. To assess cell viability, MTS [3-(4,5-dimethylthiazol-2-yl)-5-(3-carboxym-ethoxyphenyl)-2-(4-sulfophenyl)-2H-tetrazolium; inner salt] assay was performed using a CellTiter 96 AQueous assay kit (Promega Corp.).

#### EGF stimulation assay

HSC-2 and SAS cells were washed with DMEM lacking FBS to eliminate the growth factors present in the enriched medium. Then, the cells were plated in 96-well culture plates at a density of 2,000 cells per well in 100 µl of 0.1% dialyzed FBS with or without 50 ng/ml of EGF (PeproTech). MTS assay was performed after 12, 24, and 36 h.

#### Antitumor activity of Anti-EGFR antibodies

Thirty-two six-week-old female BALB/c nude mice were purchased from Charles River and used in experiments at 7 weeks of age. HSC-2 or SAS (0.3 ml of 1.33×10^8^/ml in RPMI) was mixed with 0.5 ml of BD Matrigel Matrix Growth Factor Reduced (BD Biosciences). A 100-µl suspension (containing 5×10^6^ cells) was injected subcutaneously into the left flanks of nude mice. After day 1, 100 µg of EMab-17 and control mouse IgG (Sigma-Aldrich Corp.) in 100 µl of PBS was injected into the peritoneal cavity of each mouse, followed by additional antibody injections on days 7 and 14. Mice were monitored for health and weight every 3 or 4 days. The diameter and volume of the tumor were determined as previously described (18), and the mice were euthanized 21 days after cell implantation. The duration of the experiment was three weeks. A body weight loss exceeding 25% of total body weight was defined as a humane endpoint. All data was expressed as mean ± SEM, and statistical analysis was conducted using Tukey-Kramer's test; P<0.05 was considered to indicate a statistically significant difference.

#### Statistical analyses

Statistical analysis was conducted using ANOVA followed by Tukey-Kramer's test. P<0.05 was considered to indicate a statistically significant difference. All data was expressed as mean ± SEM and analyzed using GraphPad Prism 6 (GraphPad Software, Inc.).

## Results

### 

#### Production and characterization of Anti-EGFR mAbs

In this study, one mouse was immunized with LN229/EGFR, and culture supernatants of hybridoma were screened for binding to purified EGFRec using ELISA. Flow cytometry was used as a second screening to assess reactions with LN229 and LN229/EGFR cells. LN229 cells express endogenous EGFR (15); therefore, a stronger reaction against LN229/EGFR was required. One clone was obtained-EMab-17 of IgG_2a_ subclass-although almost all mAbs were determined to be a mouse IgG_1_ subclass like EMab-51 (15) or EMab-134 (12).

**Figure 1.**
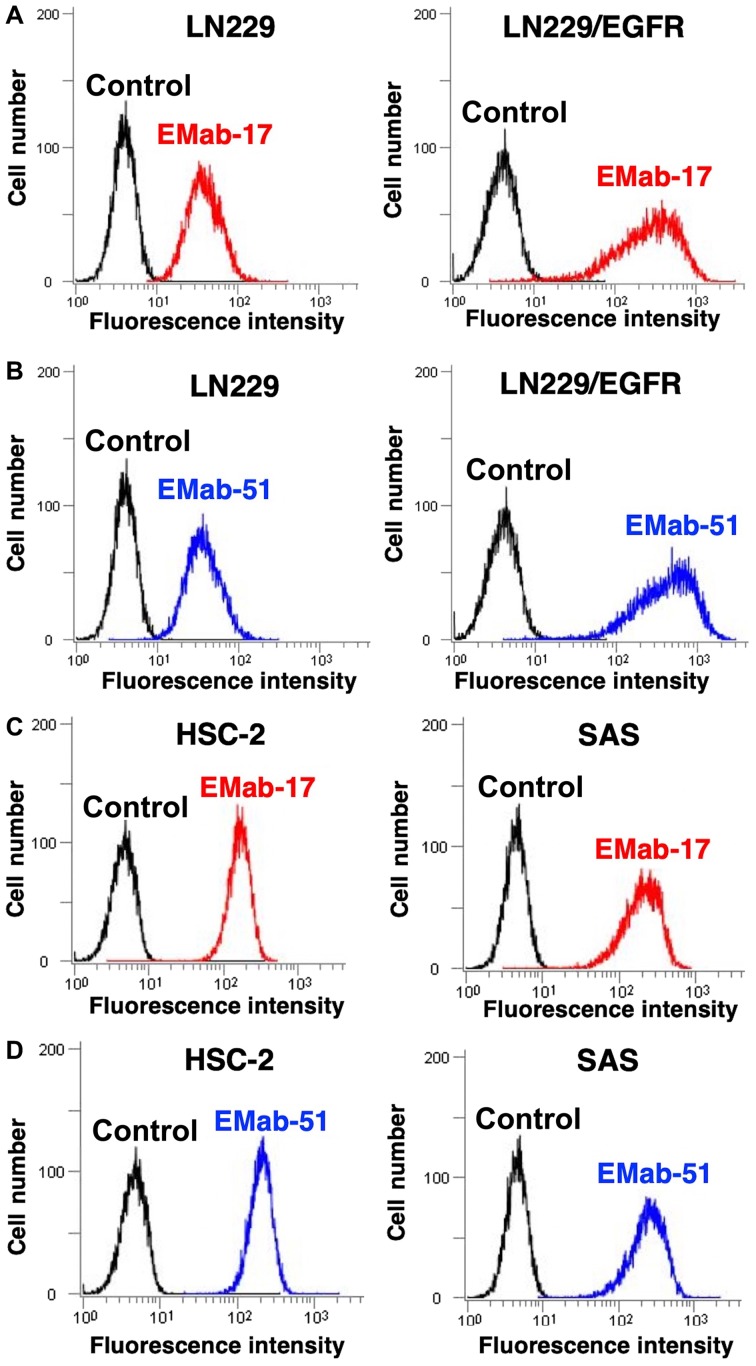
Flow cytometry of EMab-17. (A) LN229 and LN229/EGFR cells were treated with 1 µg/ml (A) EMab-17 (red line) and (B) EMab-51 (blue line) followed by Alexa Fluor 488-conjugated anti-mouse IgG. (C) HSC-2 and SAS were treated with 1 µg/ml of (C) EMab-17 (red line) and (D) EMab-51 (blue line), followed by Alexa Fluor 488-conjugated anti-mouse IgG. Black line, PBS treated control.

**Figure 2.**
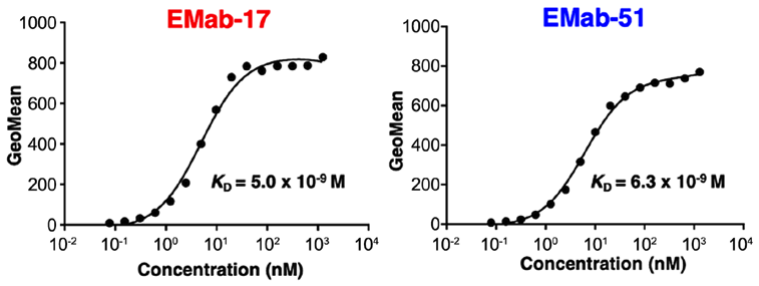
Binding affinities of anti-EGFR antibodies were determined using flow cytometry. SAS cells were suspended in 100 µl serially diluted EMab-17 or EMab-51 (0.006–100 µg/ml), after which secondary antibodies were added. Fluorescence data was collected using a cell analyzer. GeoMean, geometric mean of fluorescence intensity.

Flow cytometry was used to demonstrate a stronger reaction with EMab-17 LN229/EGFR than with endogenous EGFR-expressing LN229 brain tumor cells (Fig. 1A), which indicated that EMab-17 is EGFR-specific. As a positive control, EMab-51 demonstrated a similar reaction with LN229 and LN229/EGFR (Fig. 1B). Endogenous HSC-2 and HSC-3 OSCC cell lines were also identified with both EMab-17 and EMab-51 (Fig. 1C and D). Flow cytometry was again applied to determine the binding affinity of EMab-17 for SAS cells (Fig. 2) and calculated *K*_D_ values for EMab-17 of 5.0×10^−9^ M against SAS. Similarly, *K*_D_ values were determined for EMab-51 as 6.3×10^−9^ M against SAS (Fig. 2), revealing that both EMab-17 and EMab-51 possess a high affinity for EGFR-expressing cell lines.

#### ADCC and CDC activities against OSCC cell lines

**Figure 3.**
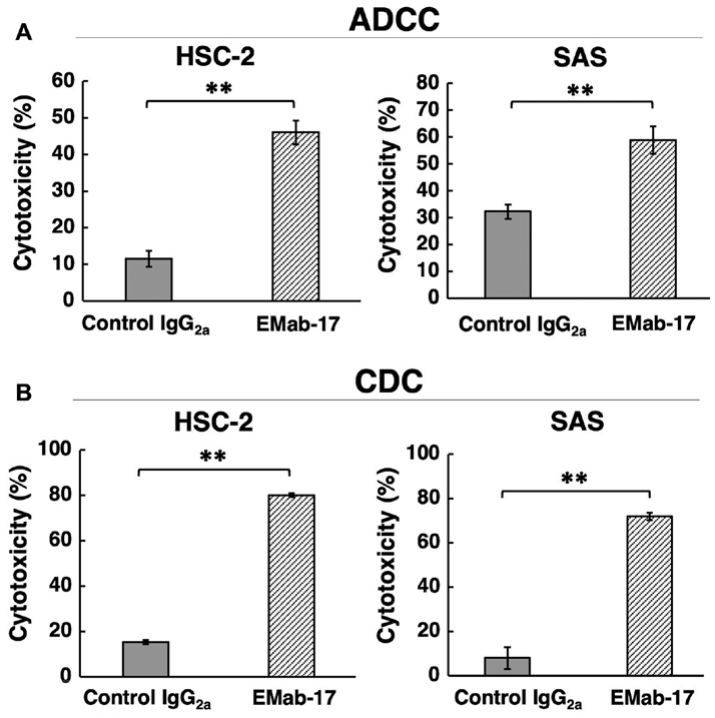
ADCC and CDC activities. For ADCC activity, cells were incubated with splenocytes from mice that were treated with the indicated antibodies at 100 µg/ml for 4 h. Cell lysis was determined using Calcein AM. For CDC activity, cells were incubated with 10% rabbit complement in the presence of the indicated antibodies for 5 h. Cell lysis was determined using an MTS assay. (A) ADCC against HSC-2 and SAS. (B) CDC against HSC-2 and SAS. **P<0.01 as indicated. ADCC, antibody-dependent cellular cytotoxicity; CDC, complement-dependent cytotoxicity.

This study examined whether EMab-17 induced ADCC and CDC in EGFR-expressing OSCC cell lines. EMab-17 was determined to be a mouse IgG_2a_ subclass that might possess both ADCC and CDC (although mouse IgG_1_ such as EMab-51 and EMab-134 does not) (12,15). As detailed in Fig. 3A, EMab-17 exhibited high ADCC activity against HSC-2 and SAS. Furthermore, high CDC activity was also observed for HSC-2 and SAS by EMab-17 (Fig. 3B), suggesting that EMab-17 might exert antitumor activities *in vivo*. Although we added EGF to SAS and HSC-2, these cell lines did not grow well compared to control cells by responding to EGF stimulation (data not shown), indicating that EMab-17 could not neutralize EGF-EGFR axis.

#### Antitumor activities against OSCC

**Figure 4.**
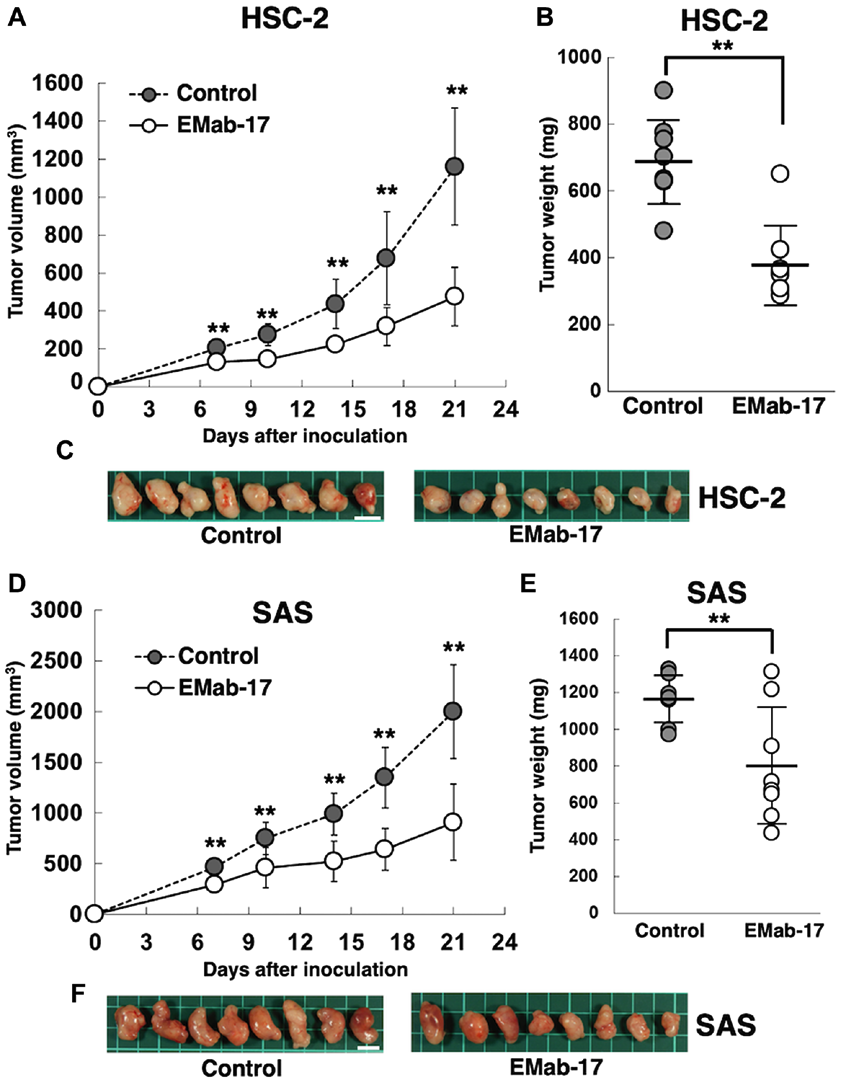
Antitumor activity of EMab-17 against HSC-2 and SAS. (A) The tumor volumes of HSC-2 ×enografts were recorded. HSC-2 cells were subcutaneously injected into female nude mice. The indicated antibodies (100 µg/day; 5 mg/kg) were administered intraperitoneally 1, 7 and 14 days after cancer cell inoculation. Tumor volume was measured at the indicated time points. (B) Tumor weight of HSC-2 ×enografts (day 21). (C) Comparison of HSC-2 tumor size (day 21). Scale bar, 1 cm. (D) Tumor volume of SAS xenografts. SAS cells were injected subcutaneously into female nude mice. The indicated antibodies (100 µg/day; 5 mg/kg) were administered intraperitoneally 1, 7 and 14 days after cancer cell inoculation. Tumor volume was measured at the indicated time points. (E) Tumor weight of SAS xenografts (day 21). (F) Comparison of SAS tumor size (day 21). Scale bar, 1 cm. Data are presented as the mean ± standard error of the mean. **P<0.01 vs. the control group or as indicated.

HSC-2 cells were subcutaneously implanted into the flanks of nude mice in order to study the antitumor activity of EMab-17 on cell growth *in vivo*. EMab-17 and the control mouse IgG were injected (on days 1, 7, and 14 after the cell injections) three times into the peritoneal cavity. Tumor formation was observed in mice from the control and EMab-17-treated groups in HSC-2 ×enograft models. EMab-17 demonstrated significant reduction in tumor development of the HSC-2 ×enograft compared with that in the control mouse IgG group on days 7, 10, 14, 17, and 21 (Fig. 4A). Mice treated with EMab-17 had significantly lower tumor weights compared to the control mouse IgG group in HSC-2 ×enograft models (Fig. 4B). The resected tumors of HSC-2 ×enografts are shown in Fig. 4C. The body weights of the HSC-2 ×enograft mice were recorded for 21 days (Fig. S1A). Body weight did not vary significantly between the two groups in the HSC-2 ×enograft models.

Identical experiments were performed using SAS xenograft models. EMab-17 demonstrated significantly reduced tumor development in the SAS xenograft group compared with that in the control mouse IgG group on days 7, 10, 14, 17, and 21 (Fig. 4D). The tumor weight of EMab-17-treated mice was significantly lower compared to the control mouse IgG group in the SAS xenograft models (Fig. 4E); the resected tumors of the SAS xenografts are illustrated in Fig. 4F. The body weights of the SAS xenograft mice were recorded for 21 days (Fig. S1B). The body weight did not vary significantly between the two SAS xenograft model groups.

Combined results confirm that EMab-17 exerted an antitumor activity against HSC-2 and SAS xenograft models via ADCC and CDC activities.

## Discussion

EGFR is the first receptor target using mAbs that has been developed for cancer treatment (19–21). This includes necitumumab (a fully human mAb; IgG_1_) for non-small cell lung cancers, panitumumab (a fully human mAb; IgG_2_) for colorectal cancers, and cetuximab (a human-mouse chimeric mAb; IgG_1_) for colorectal, head, and neck cancers. Anti-EGFR mAbs possess several functional mechanisms, including ADCC, CDC, blocking dimerization, and EGFR endocytosis. For investigating ADCC and CDC in mouse xenograft models, the subclass of mAbs should be IgG_2a_ or IgG_2b_ of mouse IgG (22), IgG_2a_ or IgG_2b_ of rat IgG (23), IgG_1_ of human IgG (24), or type B of canine IgG (25).

For the previous production of anti-EGFR mAbs (such as EMab-51 (15) or EMab-134 (12)), purified recombinant EGFR was immunized into mice. This methodology using cancer cell lines (such as LN229) for producing immunogen was previously identified as a CasMab method (26). Because almost all mAbs were determined to be a mouse IgG_1_ subclass like EMab-51 (15) or EMab-134 (12), it was not possible to investigate the antitumor activities of anti-EGFR mAbs that were produced using CasMab methods. Therefore, EMab-17 is the first anti-EGFR mAb of IgG_2a_ or IgG_2b_ that has been developed using the CasMab method.

In the present study, we added EGF to HSC-2 and SAS cell lines; however, these cell lines did not respond to EGF stimulation and did not grow well (data not shown), indicating that EMab-17 could not neutralize EGF-EGFR axis. Taken together, anti-tumor activities by EMab-17 were exerted by ADCC and CDC activities.

Unfortunately, EMab-17 was not suitable for Western blot and immunohistochemical analyses (data not shown); therefore, EMab-51 or EMab-134 should be used for diagnosing EGFR expression in cancer patients. In subsequent future studies, the subclasses of EMab-51 and EMab-134 will be converted into the mouse IgG_2a_ subclass, and comparisons between ADCC/CDC activities and EMab-17 will be made.

In conclusion, this study successfully developed an anti-EGFR mAb of an IgG_2a_ subclass-EMab-17-which demonstrated the antitumor activity via ADCC/CDC activities. EMab-17 could potentially be used for antibody-based therapy for EGFR-expressing OSCC.

## Supplementary Material

Supporting Data

## Data Availability

The datasets used and/or analyzed during the present study are available from the corresponding author on reasonable request.

## Abbreviations

EGFR: epidermal growth factor receptor
HER: human epidermal growth factor receptor
OSCC: oral squamous cell carcinoma
mAbs: monoclonal antibodies
ADCC: antibody-dependent cellular cytotoxicity
CDC: complement-dependent cytotoxicity
HNC: head and neck cancer
PD-1: programmed cell death-1
VEGF: vascular endothelial growth factor
DMEM: Dulbecco's Modified Eagle's medium
ELISA: enzyme-linked immunosorbent assay
FBS: fetal bovine serum
EDTA: ethylenediaminetetraacetic acid
BSA: bovine serum albumin
PBS: phosphate-buffered saline
