# Supplementary material for: A novel anti-EGFR monoclonal antibody (EMab-17) exerts antitumor activity against oral squamous cell carcinomas via antibody-dependent cellular cytotoxicity and complement-dependent cytotoxicity
Source: Oncol Lett. 2020 Feb 10;19(4):2809–16. doi: 10.3892/ol.2020.11384 (PMC7068343; doi:10.3892/ol.2020.11384)

Figure S1. Body weight of HSC-2 and SAS xenografts. (A) Body weight of HSC-2 xenograft mice was measured for 21 days. The values are means ± SEM. (B) Body weight of SAS xenograft mice was measured for 21 days. The values are means ± SEM.

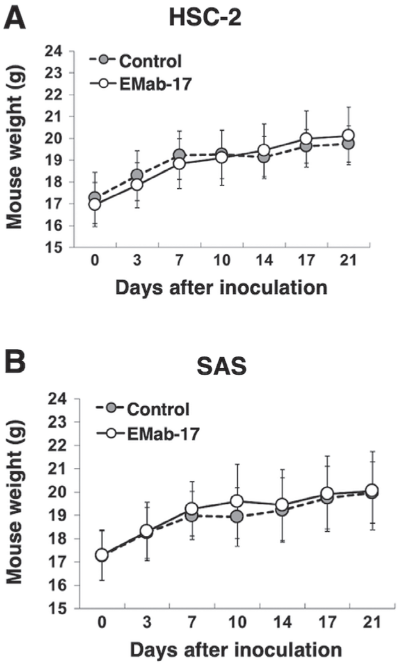

Supplement: Supporting Data [file Supplementary_Data.pdf]
